# Effectiveness of warm needling acupuncture for pain relief in patients with diabetic peripheral neuropathy: A protocol of systematic review

**DOI:** 10.1097/MD.0000000000023077

**Published:** 2020-11-06

**Authors:** Li-qin Wang, Fei Wang, Xue-hui Wang

**Affiliations:** a Department of First Clinical Medicine; b Department of Respiratory Medicine, First Affiliated Hospital of Heilongjiang University of Chinese Medicine, Harbin, China.

**Keywords:** acupuncture, diabetic peripheral neuropathy, effectiveness, moxibustion, pain, safety

## Abstract

**Background:**

Warm needling acupuncture (WNA) has been widely utilized for pain management in patients with diabetic peripheral neuropathy (DPN). However, its results are still inconsistent, and no systematic review has specifically addressed this issue. Thus, this systematic review will comprehensively and systematically investigate the effectiveness and safety of WNA for pain relief in DPN.

**Methods:**

A comprehensive literature search of MEDLINE, EMBASE, Cochrane Library, Web of Science, Scopus, Allied and Complementary Medicine Database, CBM database, and China National Knowledge Infrastructure will be performed for randomized controlled trials that report WNA for pain relief in patients with DPN. All electronic databases will be searched from initial to the present without limitations of language and publication status. Two investigators will independently screen papers, collect data, and assess study quality. Cochrane risk of bias tool will be used for study quality assessment, and evidence quality will be evaluated using Grading of Recommendations Assessment, Development and Evaluations approach. RevMan 5.3 software will be applied for running statistical analysis.

**Results:**

This study will summarize the evidence for the effectiveness and safety of WNA for the management of pain in patients with DPN.

**Conclusions:**

The findings of this study may provide helpful evidence to judge whether WNA for pain relief in DPN is effective or not.

## 1 Introduction

Diabetic peripheral neuropathy (DPN) is a common complication of diabetes mellitus (DM), affecting 50% to 90% of people with DM.^[1–3]^ It is characterized by varying degrees of pain, tingling, numbness, burning, sharp, and shooting in the extremities^[4,5]^; and is usually considered as much worse and severer at night.^[4,5]^ Of those, pain is a constant distress.^[6–9]^ It accounts for 10% to 30% of such population based on the different studied patients.^[10–17]^ If it cannot be treated effectively and timely, it can significantly restrict recreational social activities, negatively impact mood, and substantially affect quality of life in patients with DPN.^[18–20]^

The pathogenesis of pain in DPN is still not fully understood, which presents a great therapeutic challenge.^[21]^ Alternative therapy, such as acupuncture, is commonly utilized in the clinical practice of pain management with increasing popularity and is gaining acceptance among the public and academic community around the world.^[22–24]^ It is a meridian-based therapy, with needles inserting into specific acupoints on the body, which exerts distinct therapeutic actions.^[25,26]^ Clinical and animal studies found that acupuncture treatment is associated with an increase in blood circulation for DPN.^[27]^ Specifically, acupuncture needle stimulation with *Deqi* sensation showed substantially greater blood supply than surrounding tissue,^[28,29]^ which benefits DPN and relieves pain in DPN.^[30]^

Warm needling acupuncture (WNA) is a combination therapy of acupuncture and moxibustion, which simultaneously exerts effects of acupuncture, moxibustion, and specific acupoint stimulation.^[31]^ Studies have found that WNA demonstrates better efficacy in enhancing blood supply and pain relief than acupuncture or moxibustion alone.^[31,32]^ A variety of clinical trials reported that WNA can be widely utilized for pain relief in patients with DPN.^[33–48]^ However, all conclusions are drawn from an individual clinical trial. In addition, although 3 relevant systematic reviews have been conducted to explore the effect of acupuncture for DPN,^[49–51]^ no study specifically investigates the effectiveness and safety of WNA on pain relief in DPN systematically and comprehensively. Therefore, this systematic review protocol will specifically assess the effectiveness of WNA for pain relief in DPN.

## 2 Methods

### 2.1 Study registration

This protocol is funded and registered with osf.io/m4qe3. It has been reported based on the guideline of Preferred Reporting Items for Systematic Review and Meta-Analysis Protocols statement.^[52]^

### 2.2 Eligibility criteria

#### 2.2.1 Inclusion criteria

Inclusion criteria are as follows: Types of studies: only randomized controlled trials (RCTs) on use of WNA for pain relief in patients with DPN; Types of interventions: studies including any types of WNA, regardless duration, frequency, dosage, and other related parameters; Types of controls: all comparators are eligible for inclusion, such as pain killers, acupuncture or moxibustion alone; Types of participants: only adult patients (≥18 years old) with confirmed diagnosis of painful DPN, irrespective race, gender, medical histories, and conditions; and Types of outcomes: at least 1 outcome indicator related to DPN pain.

#### 2.2.2 Exclusion criteria

Exclusion criteria are as follows: Besides WNA, treatments also involved nonpenetrating needles, electroacupuncture, acupuncture, or moxibustion in the intervention group; comparator involved any forms of WNA; outcome indicators without pain measurement; any other studies, such as animal study, review, case report, case series, comments, nonclinical trial, uncontrolled study, and non-RCTs; and repeated or overlapping publications.

#### 2.2.3 Outcome measurements

The primary outcome is pain intensity, as measured by any pain scales, such as Visual analogue scale, Numeric Rating Scale, or any other relevant tools.

The secondary outcomes consist of sensory conduction velocity, motor conduction velocity of the peroneal nerve, complete response rate, neuropathic syndrome score, glycemic profile, quality of life (as assessed by any associated scales), and adverse events.

### 2.3 Search strategy

#### 2.3.1 Electronic databases sources

A comprehensive literature search for relevant randomized controlled trials will be conducted from their initial to the present in the following electronic databases: MEDLINE, EMBASE, Cochrane Library, Web of Science, Scopus, Allied and Complementary Medicine Database, CBM database, and China National Knowledge Infrastructure. We will not apply any limitations to language and publication status. A research librarian with expertise in literature search strategies will help to develop detailed sample for MEDLINE (Table 1). We will also create similar detailed search strategies for other electronic databases.

**Table 1:** Search strategy applied to MEDLINE.

Number	Search terms
1	Diabetic peripheral neuropathy
2	Diabetic neuropathy
3	Peripheral neuropathy
4	Distal symmetric peripheral neuropathy
5	Diabetes
6	Diabetic mellitus
7	Pain
8	Painful
9	Pain intensity
10	Pain relief
11	Or 1–10
12	Acupuncture
13	Warm needle
14	Needling
15	Electroacupuncture
16	Acupoint
17	Scalp acupuncture
18	Manual acupuncture
19	Moxibustion
20	Or 12–19
21	Randomized controlled trials
22	Clinical trials
23	Controlled study
24	Random
25	Randomly
26	Blind
27	Concealment
28	Placebo
29	Control
30	Comparator
31	Study
32	Trial
33	Or 21–32
34	11 and 20 and 33

#### 2.3.2 Other literature sources

We will plan to search gray literatures, such as conference proceedings, websites of clinical trial registries, and reference lists of all included studies.

### 2.4 Study selection

All relevant citations will be selected through a 2-stage process by 2 independent investigators, respectively. In the first stage, titles and abstracts of all citations will be screened, and those considered not to be associated with the topic will be excluded. In the second stage, 2 investigators will examine the remaining full papers for concordance to check whether they fulfill all inclusion criteria. A third experienced investigator will help to solve any disagreements between 2 investigators. The flowchart with results generated and reasons for exclusion will be presented.

### 2.5 Data collection and management

Two investigators will independently collect data from each included trial using a structured standardized form. Any disagreements in the data collection between 2 investigators will be solved by a third experienced investigator. Data collection includes authors, year of publication, region, number of patients, patient demographics (gender, age, diagnostic criteria, and eligibility criteria), study methods (details of randomization, blind, and allocation), intervention and control details (delivery types, mode, frequency, dosage, and duration), outcome measurements, and follow-up details. If we identify any insufficient or missing information, we will ask for primary authors to provide that. If that kind of information is not available, we will only analyze data at hand using intention-to-treat analysis, and will discuss its potential affects.

### 2.6 Study quality assessment

Study quality for each included trial will be evaluated using Cochrane risk of bias tool by 2 independent investigators, respectively. It covers domains of selection bias, performance bias, attrition bias, detection bias, reporting bias, and other bias. Each field will be further graded as high, unclear or low risk of bias. Disagreements will be solved by a third investigator through discussion.

### 2.7 Statistical analysis

#### 2.7.1 Data synthesis

We will employ RevMan 5.3 software to analyze extracted data, and carry out a meta-analysis whenever it is possible. We will estimate outcomes as relative risks, odds ratios, or risk difference and 95% confidence intervals (CIs) for dichotomous data, and weighted mean difference or standardized mean difference and 95% CIs for continuous data. The data from each trial will be utilized to construct evidence tables of a narrative summary description of the included trials. If the trials are diverse and quantitative synthesis is not feasible, we will report trials using albatross plots based on the methodological guideline.^[53]^ Statistical heterogeneity across trials with respect to characteristics of studies will be identified by *I*^2^ test.^[54]^*I*^2^ ≤ 50% exerts little statistical heterogeneity, and a fixed-effects model will be applied. *I*^2^ > 50% indicates distinct heterogeneity, and a random-effects model will be placed.^[55]^ If meta-analysis is appropriate and trials are found to be combinable, fixed-effects or random-effects meta-analysis will be carried out, and will calculate pooled effect sizes for primary and secondary outcomes. We will conduct it when at least 2 eligible trials available for comparison and they are sufficiently homogeneous in terms of study design, participants, interventions, and outcomes. For all outcomes, no quantitative synthesis will be undertaken and coefficients of each reported outcome will be elaborated at the study level.

#### 2.7.2 Subgroup analysis

If necessary, we will undertake a subgroup analysis based on the different patient demographics, study quality, types of interventions and comparators, and outcome measurements.

#### 2.7.3 Sensitivity analysis

We will plan to conduct sensitivity analysis to verify the stability of conclusions by deleting low-quality studies.

#### 2.7.4 Reporting bias

If at least 10 eligible trials are included, we will explore the reporting bias using funnel plot and Egger regression test.^[56,57]^

### 2.8 Grading the quality of evidence

Quality of evidence will be checked by 2 investigators according to the Grading of Recommendations Assessment, Development and Evaluation. It covers 4 different levels, including very low, low, moderate, and high levels.^[58]^ Any conflicts will be cleared up by discussion with a third investigator, and a final consensus will be reached.

### 2.9 Ethics and dissemination

No ethical approval is required, because no individual patient data will be obtained in this study. This study is expected to be disseminated through a peer-reviewed journal.

## 3 Discussion

Painful DPN is a common disorder in patients with DM, which brings very poor quality for such patients. Presently, WNA approach is utilized for the treatment of pain in DPN. In spite of the clinical and experimental support, the effect and safety of WNA for pain have not been fully validated and evaluated. In addition, no systematic review and meta-analysis regarding it has been conducted. Therefore, this study aims to assess the effect and safety of WNA for pain in DPN. The results of this study have the potential influence on the management of WNA for pain relief in DPN. Its findings may supply evidence for the reference of clinical practice and health-related policy maker.

## Author contributions

**Conceptualization:** Li-qin Wang, Fei Wang, Xuehui Wang.

**Data curation:** Li-qin Wang, Fei Wang, Xuehui Wang.

**Formal analysis:** Li-qin Wang, Fei Wang, Xuehui Wang.

**Funding acquisition:** Li-qin Wang, Xuehui Wang.

**Investigation:** Xuehui Wang.

**Methodology:** Fei Wang.

**Project administration:** Xuehui Wang.

**Resources:** Li-qin Wang, Fei Wang.

**Software:** Li-qin Wang, Fei Wang.

**Supervision:** Xuehui Wang.

**Validation:** Li-qin Wang, Fei Wang, Xuehui Wang.

**Visualization:** Li-qin Wang, Fei Wang, Xuehui Wang.

**Writing – original draft:** Li-qin Wang, Xuehui Wang.

**Writing – review & editing:** Li-qin Wang, Fei Wang, Xuehui Wang.
